# Coordinating Regulation of Gene Expression in Cardiovascular Disease: Interactions between Chromatin Modifiers and Transcription Factors

**DOI:** 10.3389/fcvm.2017.00019

**Published:** 2017-04-06

**Authors:** Ashley J. Bauer, Kathleen A. Martin

**Affiliations:** ^1^Department of Medicine (Cardiovascular Medicine), Cardiovascular Research Center, Yale University School of Medicine, New Haven, CT, USA; ^2^Department of Pharmacology, Cardiovascular Research Center, Yale University School of Medicine, New Haven, CT, USA

**Keywords:** epigenetics, transcription factors, chromatin modifiers, cardiovascular disease, gene expression

## Abstract

Cardiovascular disease is a leading cause of death with increasing economic burden. The pathogenesis of cardiovascular diseases is complex, but can arise from genetic and/or environmental risk factors. This can lead to dysregulated gene expression in numerous cell types including cardiomyocytes, endothelial cells, vascular smooth muscle cells, and inflammatory cells. While initial studies addressed transcriptional control of gene expression, epigenetics has been increasingly appreciated to also play an important role in this process through alterations in chromatin structure and gene accessibility. Chromatin-modifying proteins including enzymes that modulate DNA methylation, histone methylation, and histone acetylation can influence gene expression in numerous ways. These chromatin modifiers and their marks can promote or prevent transcription factor recruitment to regulatory regions of genes through modifications to DNA, histones, or the transcription factors themselves. This review will focus on the emerging question of how epigenetic modifiers and transcription factors interact to coordinately regulate gene expression in cardiovascular disease. While most studies have addressed the roles of either epigenetic or transcriptional control, our understanding of the integration of these processes is only just beginning. Interrogating these interactions is challenging, and improved technical approaches will be needed to fully dissect the temporal and spatial relationships between transcription factors, chromatin modifiers, and gene expression in cardiovascular disease. We summarize the current state of the field and provide perspectives on limitations and future directions. Through studies of epigenetic and transcriptional interactions, we can advance our understanding of the basic mechanisms of cardiovascular disease pathogenesis to develop novel therapeutics.

## Introduction

Cardiovascular disease is the leading cause of death in the United States and worldwide (1). More than one in three adults in the United States has one or more types of cardiovascular diseases, which include coronary heart disease, high blood pressure, congenital heart defects, and heart failure, among others (1). In addition to health burdens, cardiovascular disease generates economic burdens greater than $316 billion dollars per year in the United States and projected to reach $918 billion by 2030 and over one trillion dollars worldwide (1). Numerous risk factors, both heritable and behavioral, contribute to cardiovascular disease. These include genetics, high blood pressure, high cholesterol, diabetes, smoking, obesity, physical inactivity, and others (1).

The multiple cell types that make up the heart and vasculature can contribute to cardiovascular disease in different ways. The heart is made up of cardiac myocytes and fibroblasts and is supplied by an extensive network of blood vessels, which in turn are composed of multiple cell types including fibroblasts and connective tissue, smooth muscle cells that regulate vessel tone, and the endothelial cells (ECs) that line the vessel and are in direct contact with the blood and its cellular components. Cardiovascular diseases can arise from numerous factors including structural heart and vascular defects, inflammatory responses, and endothelial and vascular smooth muscle dysfunction. Dysfunction at the tissue level can arise from different types of molecular pathologies such as genetic mutations and impaired signaling mechanisms. These changes are ultimately manifest at the level of gene expression. Traditionally, investigation into underlying mechanisms has been mediated at the level of transcription of genes into mRNA and, ultimately, to protein. More recently, it has been appreciated that epigenetics provides a major influence on gene expression and cell function.

Epigenetics is defined as a stably heritable phenotype resulting from changes in a chromosome without alterations in the DNA sequence (2). While non-coding RNAs are also epigenetic mediators, this review will focus on enzymatic epigenetic regulators. Each chromosome is made up of DNA and associated proteins. The complex of DNA wrapped around octamers of histone proteins is known as chromatin, which functions to package and compact DNA into each nucleus. The structural and functional subunit of chromatin is the nucleosome, which is composed of eight histone proteins (two of each of the histones H2A, H2B, H3, and H4) that form an octamer that binds approximately 146 base pairs of DNA (3). Covalent modifications can be made to histone tails or the DNA itself. These include DNA modifications such as cytosine methylation [5-methylcytosine (5mC)] or posttranslational modification of histone tails including acetylation or methylation (4). Cytosine methylation at CpG dinucleotides is typically considered a repressive modification associated with inactive chromatin. The 5mC modification is generated *de novo* by the DNA methyltransferases, DNMT3a and DNMT3b, while DNA methylation is maintained during replication by DNMT1 using the already methylated DNA strand as a template. 5mC can be converted to 5-hmC by the ten–eleven translocation (TET) proteins, an α-ketoglutarate-dependent family of oxidases. 5-hmC can not only serve as a stable mark allowing for activation of gene expression but also serve as an intermediate step to DNA demethylation through the base excision repair pathway (5). In addition to DNA modifications, posttranslational histone tail modifications can serve as a repressive or activating signal depending on the site on the histone tail being modified, number of groups added, and the region of chromatin where the modification occurs (for example, promoter versus intergenic regions). Acetylation of histone tails is an important epigenetic regulation that adds negative charge to histones and is typically characteristic of transcriptionally active, non-compact chromatin (6). The enzymes that add and remove the acetyl group from histone tails are termed histone acetyltransferases (HATs) and histone deacetylases (HDACs), respectively. Unlike most histone tail acetylation, histone tail methylation can be activating or repressing. As with other histone tail modifications, there are numerous enzymes involved in methylating or demethylating histone tails. Common DNA and histone tail modifications and the writer or eraser enzymes that add or remove them that are discussed in this review are listed in Table 1.

**Table 1:** **List of chromatin modifiers discussed in this review and their respective roles**.

Chromatin modifier	Function	Activity	Role
p300	Histone acetyltransferase (HAT)	Acetylation of histones, non-histone proteins	Activating
CBP	HAT	Acetylation of histones, non-histone proteins	Activating
HDAC1–9, 11	Histone deacetylase (HDAC)	Deacetylation of histones, non-histone proteins	Repressive^a^
SIRT	HDAC	Deacetylation of histone, non-histone proteins	Repressive^a^
UTX (KDM6A)	Histone demethylase	Demethylation of H3K27	Activating
JMJD2a (KDM4A)	Histone demethylase	Demethylation of H3K9, H3K36	Activating
KDM3A (JMJD1A)	Histone demethylase	Demethylation of H3K9	Activation
JMJD6	Histone demethylase	Demethylation of H3R	Repressive
Protein arginine methyltransferase 4	Histone methylation	Methylation of H3R17	Activation
EZH2	Histone methyltransferase	Methylation of H3K27	Repressive
COMPASS (including ASH2, WDR5)	SET/MLL family of methyltransferases	H3K4 methylation	Activating
DNMT	DNA methylation	Methylation of DNA cytosines	Repressive
Ten–eleven translocation	DNA hydroxymethylation	Hydroxymethylation of DNA methylcytosines	Activation

*This is only a subset of the known chromatin modifying proteins and their activity. Whether there is a role for other chromatin modifying proteins in regulation of gene expression in cardiovascular disease through interaction with transcription factors is unknown*.

*^a^Reported activating role although primarily considered repressive*.

Together, these epigenetic modifications influence gene expression by altering chromatin structure (7). This can result in genes being silenced when in closed, highly compacted heterochromatin, or active in open euchromatin that is readily accessible to transcription factors. In addition to altering the compaction of the local chromatin landscape, epigenetic modifications can serve as signals to recruit other regulatory factors including transcription factors to influence gene expression. Epigenetic modifiers have been documented to interact with transcription factors in several different ways. Indirectly, the marks deposited by epigenetic regulators can recruit or prevent transcription factor binding. More directly, these epigenetic modifiers can be recruited to their target genes by transcription factors. The epigenetic enzymes then modify histone tails or DNA in this targeted region, or in some instances, they can be prevented from binding to chromatin by transcription factors. Moreover, epigenetic modifiers can posttranslationally modify the transcription factors themselves, which subsequently alters the activity and/or binding of these transcription factors to the DNA or to other chromatin modifying proteins. Various histone-modifying enzymes and their marks have been implicated in the pathogenesis of cardiovascular disease (8–13), and the importance of their complex interactions with transcription factors is increasingly being investigated and better understood. Our focus in this review is to describe examples of these interactions that occur in settings of cardiovascular disease and highlight questions that remain to be investigated.

Although few studies have investigated the role of these interactions in cardiovascular disease, it is essential to elucidate how transcription factors and chromatin modifiers interact to understand cardiovascular disease pathology at a basic level. By understanding these interactions, we will be able to, ultimately, develop novel therapeutics to treat cardiovascular diseases. Although it has been established that several transcription factors implicated in cardiovascular disease pathogenesis are epigenetically regulated, this review will primarily focus on the interactions of transcription factors and DNA modifiers, histone methylation enzymes, and histone acetylation enzymes in the regulation of gene expression in various cell types and settings of cardiovascular disease.

## Histone Acetylation in Cardiovascular Disease

### Histone Acetylation and Cardiovascular Development

**Figure 1:**
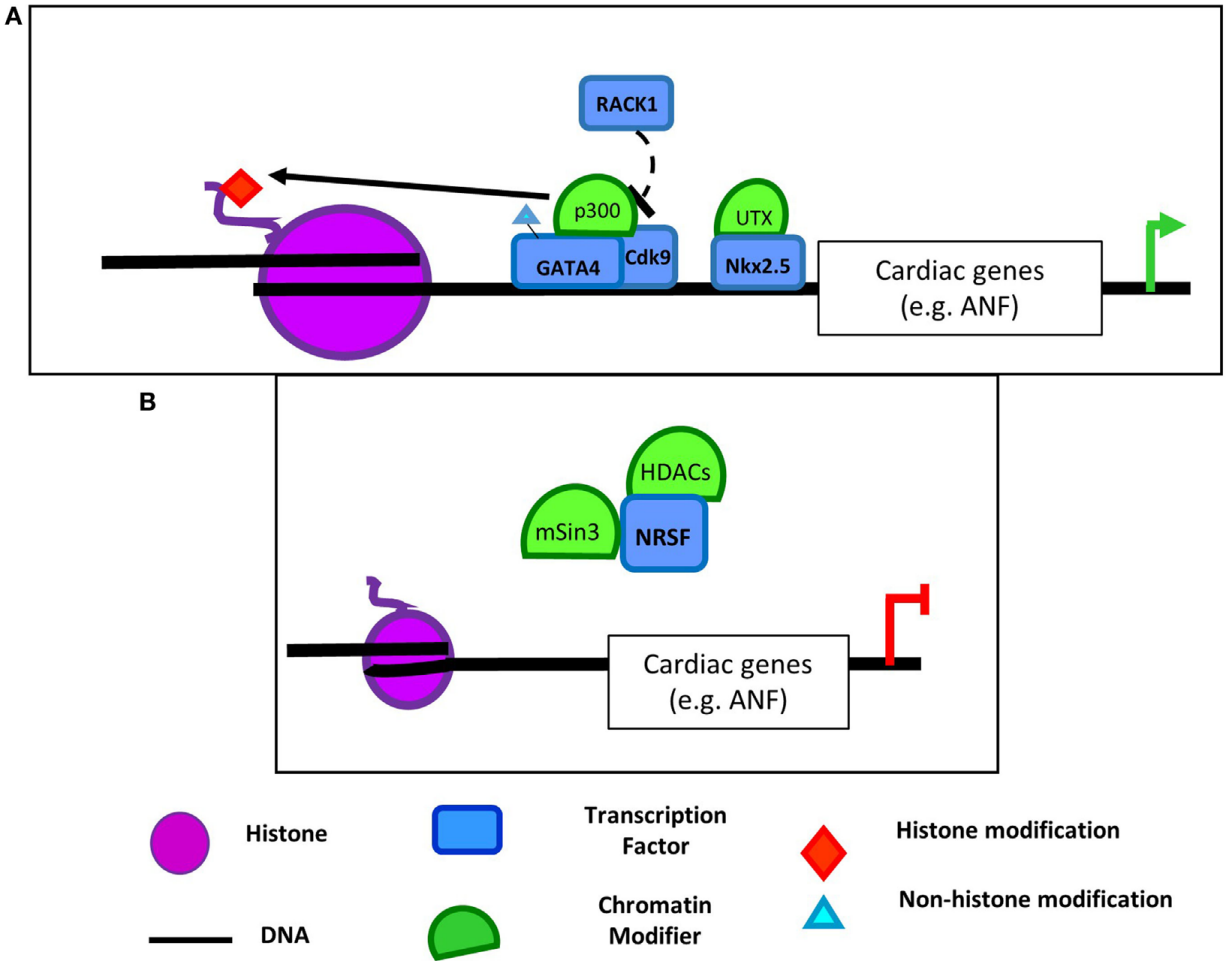
**Examples of chromatin-modifying enzymes and transcription factor complexes that interact to regulate cardiac gene expression**. **(A)** p300 acetylates GATA4 and, through interaction with Cdk9, hyperacetylates cardiac gene promoters (16). Interaction of p300, GATA4, and Cdk9 can be prevented by RACK1 (17). Nkx2.5 also interacts with the histone demethylase UTX (18). **(B)** However, neuron-restrictive silencer factor (NRSF) in a complex with mSin3 and HDAC1/2 deacetylates cardiac gene promoters resulting in repression of cardiac gene expression (19). NRSF also interacts with HDAC4/5 to repress cardiac gene expression (20), but the contexts in which NRSF interacts with each histone deacetylase (HDAC) require further elucidation.

Pathologic cardiac hypertrophy can occur in response to increased workload on the heart and is characterized by major changes in gene expression. There are distinct programs of gene expression observed in the fetal, neonatal, adult, and pathologically remodeling heart. Upregulation of fetal cardiac genes [e.g., ANF, brain natriuretic peptide (BNP)] is important in cardiac development, but their expression is generally reduced in adult cardiomyocytes. In the case of cardiomyocyte hypertrophy and hypervolemic states, their expression becomes reactivated (14, 15). Several transcription factors including GATA4 regulate cardiac genes in cardiac development and heart diseases like cardiac hypertrophy or congenital heart disease when dysregulated (14, 15). These transcription factors influence cardiac-specific gene expression not only through direct DNA binding but also through interactions with HATs and HDACs. For example, hypertrophic stimuli induce the expression of cardiac genes in cardiomyocytes through recruitment of a p300-GATA4-Cdk9 complex (Figure 1A) (16). Cdk9 is also required for p300-induced acetylation of GATA4. Conversely, the scaffold protein receptor for activated protein kinase C1 (RACK1) inhibits the hypertrophic response by preventing the interaction between GATA4 and p300 (Figure 1A) (17). These findings highlight the diverse role for p300 in being recruited to promoters to regulate gene expression as well as directly acetylating the transcription factor recruiting it. These two roles of HATs are likely not mutually exclusive and can be challenging to differentiate when identifying the role of HATs in regulation of gene expression.

Histone deacetylases also have similar complex roles in cardiovascular development and disease (21). For example, HDAC2 has opposing effects on genes involved in cardiomyocyte proliferation and hypertrophy (22, 23). HDAC2 deacetylates GATA4 to prevent its activation (22), but still promotes hypertrophic gene expression by associating with the transcription factor Ying Yang 1 (YY1) to promote activation of the fetal cardiac gene BNP in rat neonatal cardiomyocytes *in vitro* (23). However, it was not determined whether YY1 and HDAC2 co-localize at the *BNP* promoter or if knockdown of YY1 prevented HDAC2 binding to the promoter. These findings are in contrast to the activating role normally attributed to histone acetylation and the inactivating role for HDACs. However, while less common, gene activation by HDACs has been previously reported and could potentially be attributed to steric hindrance by the acetylated histone itself or another factor binding the acetylated region (24). The opposing roles for HDACs and histone acetylation are likely involved in numerous settings of cardiovascular disease.

Distinct HDAC complexes also play a more traditional role in repressing hypertrophic gene expression. In ventricular cardiomyocytes, ANF expression is repressed by the transcription factor neuron-restrictive silencer factor (NRSF) when it is associated with a mSin3-HDAC1/2 complex. Together, this complex coordinates deacetylation of NRSF-binding elements upstream of ANF (Figure 1B) (19). Moreover, NRSF also associates with HDAC4/5 to repress ANF expression, and this interaction is decreased in models of cardiac hypertrophy (20). HDACs play an important role in cardiac development and disease (25–27) as global inhibition of HDACs using pharmacologic inhibitors reduced hypertrophy in angiotensin II (AngII) and aortic banding mouse models of cardiac hypertrophy (28). However, whether these responses are dependent on their deacetylation of transcription factors, deacetylation of histones following recruitment by transcription factors to promoters, or a combination of both requires further elucidation.

### Histone Acetylation and Vascular Smooth Muscle Cells (VSMCs)

Unlike cardiac and skeletal myocytes, VSMCs do not terminally differentiate, but retain a plasticity to dedifferentiate in response to environmental cues. VSMC dedifferentiation to synthetic or inflammatory phenotypes contributes to cardiovascular pathologies including intimal hyperplasia and atherosclerosis (29, 30). Growth factors and cytokines including transforming growth factor (TGF)-β, platelet-derived growth factor (PDGF), interferon-γ (IFNγ), and interleukin (IL)-1β can dramatically alter VSMC phenotype (29, 31). Epigenetics has also been shown to play an important role in regulation of VSMC phenotype [reviewed in Ref. (11, 12)]. In general, HAT activity promotes, while HDAC activity represses smooth muscle-specific gene expression (12), which is mediated through interactions between several key transcription factors.

**Figure 2:**
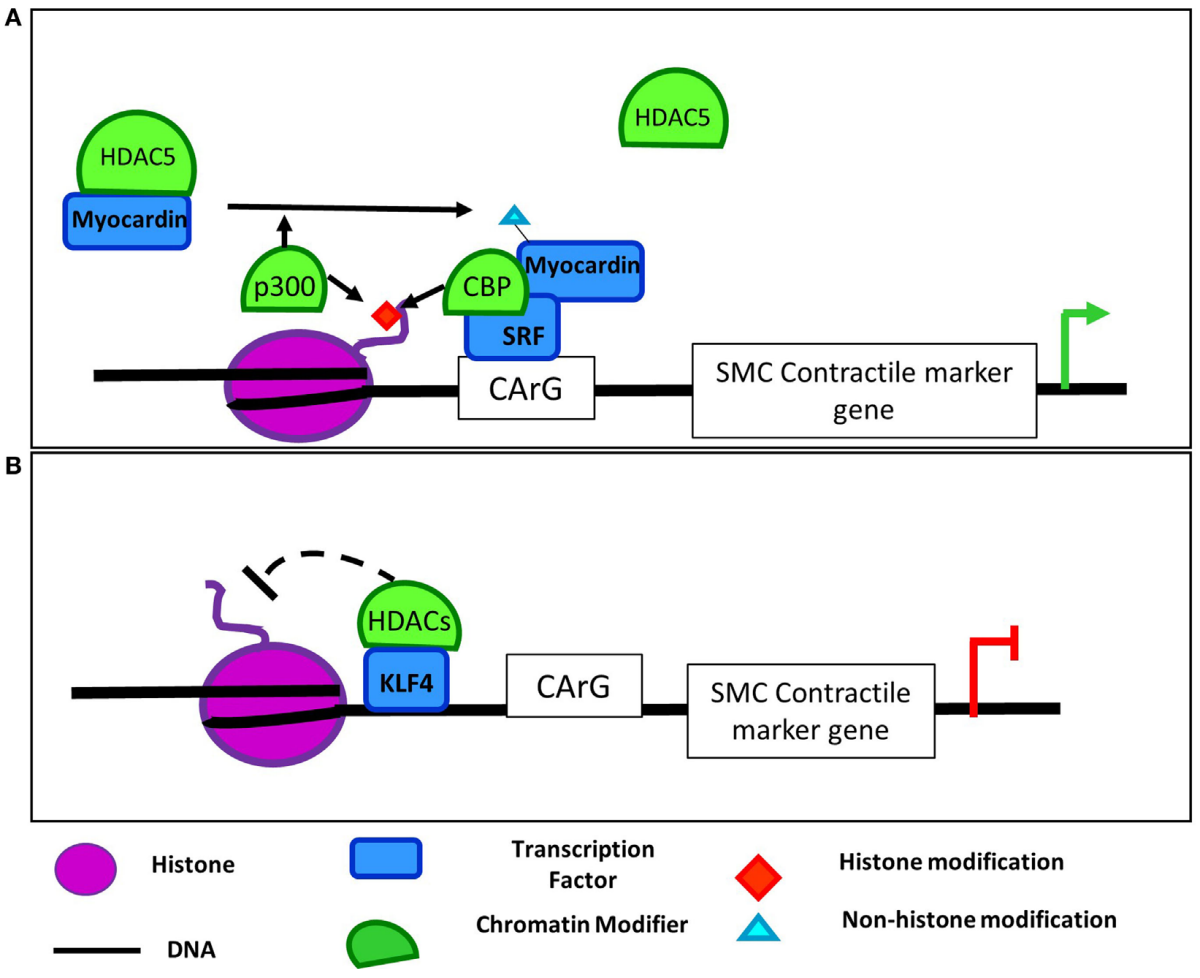
**Regulation of SMC contractile marker genes by epigenetic modifiers and transcription factors**. **(A)** p300 acetylates H3 and myocardin in SMCs promoting myocardin dissociation from HDAC5 and interaction with SRF (34, 35). CBP and SRF also interact to hyperacetylate SMC contractile marker promoters (33). Together myocardin and SRF bind to CArG boxes in SMC contractile marker promoters to induce their expression. **(B)** In contrast, dedifferentiation stimuli like platelet-derived growth factor (PDGF) promote KLF4-dependent recruitment of histone deacetylases (HDACs) 2, 4, and 5 and subsequent deacetylation of SMC marker gene promoters. (37–39). This deacetylation prevents SRF and MRTF binding to contractile genes and their activation.

Myocardin, MRTFs, and SRF are master regulators of the muscle phenotype, with roles in lineage identity and muscle-specific gene expression in cardiac, smooth, and skeletal muscle. While myocardin promotes cardiac and smooth muscle gene expression, it represses the skeletal muscle program (32). In all of these tissues, SRF binds to CArG elements in muscle-specific genes, with cofactors determining the expression of cytoskeletal and muscle-specific genes. HATs and HDACs interact with these essential transcriptional regulators. Complexes of SRF and CBP enable activation of smooth muscle contractile marker promoters like SM22α through hyperacetylation of their promoters in VSMCs (Figure 2A) (33). Enhanced activation of the SMC-specific promoters was found to be not only due to increased H3Ac (34), but also due to p300-dependent acetylation of myocardin (Figure 2A) (35). Acetylation of myocardin enhances its association with SRF and CArG boxes and is required for activation of SMC genes. Moreover, myocardin acetylation leads to its dissociation from HDAC5 (35). HDAC5 along with HDAC2 and HDAC4 deacetylate SMC marker genes downstream of PDGF-BB treatment. This inhibits binding of MRTFs and myocardin and reduces contractile gene expression (34, 36). This family of transcription factors and coactivators plays a clear role in mediating recruitment of epigenetic factors including HATs, HDACs, and histone methylating and demethylating enzymes (described in Section “Histone Methylation and Myocardin, MRTFs, and SRF Interactions”). The acetylation of myocardin itself provides another example of a transcriptional regulator that both recruits and is modified by HATs/HDACs. Given their importance in SMC phenotype, it would be of interest to determine whether the SRF cofactors, myocardin and MRTFs, similarly interact with additional epigenetic regulators.

The Kruppel-like factor (KLF) family of transcription factors play important roles in VSMC phenotypic modulation, and several studies have demonstrated a role for interactions between KLFs, p300, and HDACs in the regulation of VSMC plasticity. Specifically, KLF5, retinoic acid receptor (RAR)-α, and HDAC2 interact at the *p21* promoter to repress its expression and induce proliferation in VSMCs (40). When stimulated with a RAR-α agonist, HDAC2 deacetylates KLF5 to dissociate it from the *p21* promoter. This dissociation allowed p300 to acetylate RAR-α to stabilize its binding, activate the *p21* promoter, and block VSMC proliferation. Similarly, KLF4 can also activate gene transcription through its interaction with HATs. KLF4 associates with p300 following TGF-β1 stimulation in VSMC leading to increased H3 acetylation at TGF-β control elements of the *p21* promoter (41). TGF-β also promotes p300 interaction with Smad3 to induce acetylation of the *SM22a* promoter (42). Vascular injury, growth factors, and oxidized phospholipids can also induce KLF4 and pELK1 binding to contractile gene promoters leading to recruitment of HDAC2 and HDAC5, deacetylation of histones in these regions, inhibition of SRF binding, and repression of contractile gene expression (Figure 2B) (37–39). KLF4’s complex role in regulating VSMC phenotypic switching (43) appears to be mediated through both modifications by HATs and HDACs as well as recruitment of them to target promoters and subsequent modification of histone acetylation. Perhaps interaction of KLF4 with these epigenetic regulators could account for its context-dependent effects and may provide a useful system in which to study recruitment of epigenetic regulators to specific loci in one context, but not another.

### Histone Acetylation and ECs

Histone deacetylases and HATs also have a known role in differentiation and maintenance of EC homeostasis, including effects on proliferation, migration, and apoptosis (44). EC maintenance and angiogenesis ensure adequate blood supply to tissues, while EC inflammation is a major driver of atherosclerosis and subsequent ischemia or infarct. Vascular endothelial-cadherin (VE-cadherin) is a key component of adherens junctions and flow mechanosensing in ECs and is required for vascular integrity (45). While MRTFs and SRF have been described in terms of their roles in muscle-specific gene expression, these factors are also expressed ubiquitously in somatic cells, including ECs, where they are essential regulators of cytoskeletal genes. MRTF-A interacts with p300 in ECs to induce VE-cadherin expression (46). Overexpression of p300 enhanced vascular endothelial growth factor (VEGF)-dependent increased expression of VE-cadherin through upregulation of H3Ac and H4Ac and subsequent induction of MRTF-A recruitment to the VE-cadherin promoter in human umbilical vein endothelial cells (HUVECs) (46).

β-Catenin is another cadherin that interacts with VE-cadherin and promotes cell–cell adhesions intracellularly as a part of its structural role (47). It is also an important signaling molecule that induces gene transcription downstream of Wnt signaling (48) and is involved in angiogenesis (49). In ECs, proliferation is suppressed through downregulation of cyclin D1 when β-catenin is sequestered in the cytoplasm by direct interaction with HDAC7 (50). Upon VEGF treatment, HDAC7 is degraded and β-catenin translocates to the nucleus. Once in the nucleus, activated β-catenin upregulates proangiogenic factors like VEGF or IL-8 (51). Conversely, expression of IL-8 can be repressed by the transcription factor Bach1. Modulation of Bach1 expression alters H3 and H4 acetylation at the *IL-8* promoter, and Bach1 prevents interaction between β-catenin and p300/CBP (52). Indeed, it has previously been reported that p300-dependent histone acetylation can recruit β-catenin to enhancers during stem cell differentiation (53) and, conversely, that β-catenin can recruit p300/CBP to target genes in colon cancer cells and *Xenopus* embryos (54, 55). These findings suggest that interaction of p300/CBP and β-catenin is a common mechanism to regulate gene expression, and future studies should investigate the role of this interaction in regulation of other genes in ECs and in other settings of vascular disease.

In addition to regulation of proangiogenic factors, HATs have a role in regulating numerous transcription factors involved in EC function. p300, H3Ac, and p65 were found to be simultaneously localized to NF-kB binding sites at the *FasL* promoter, a proapoptotic factor, in ECs following antiangiogenic treatment (56). Conversely, NF-kB interacted with HDAC1, while p300, H3Ac, and H4Ac decreased at the *cFLIP* promoter, an antiapoptotic factor. This leads to repression of its expression indicating that NF-kB interaction with both HDACs and HATs is important in regulating EC function through changes in histone acetylation at numerous EC genes. Indeed, VCAM-1 expression is also activated when p300 and NF-kB p65 directly bind to the VCAM-1 promoter in ECs and acetylate H3K9 and H4K8 (57). In addition to interacting with NF-kB at gene promoters, p300 also acetylates p65 itself to promote its activation (57). This also leads to induction of VCAM-1 expression, an important adhesion molecule. Acetylation of p65 by p300 is prevented through phosphorylation of p300 by AMPK overexpression. AMPK becomes activated in response to numerous stresses including nutrient deprivation and inflammation, and it has been reported to have a vasculoprotective role in the endothelium (58). It is likely that p300 (and potentially CBP) has the dual role of histone acetylation through recruitment by a transcription factor in addition to acetylation of that transcription factor itself in regulation of gene expression in settings in addition to those presented throughout this review.

p300 regulation of NF-kB is also involved in activation of the endothelial nitric oxides synthase (eNOS) promoter, the major source of NO in response to acute laminar shear stress, which is an important protective process in normal EC function and signaling (59). EC response to flow is highly dependent on the amount, duration, and direction of flow (60, 61). High laminar shear stress transiently upregulates NF-kB and pro-inflammatory pathways, increases eNOS expression, aligns ECs in the direction of flow, and promotes an atheroprotective EC phenotype (62). Conversely, disturbed flow leads to sustained expression of pro-inflammatory pathways, a failure of EC alignment, and a proatherogenic phenotype. It is reported that NF-kB activates the eNOS promoter with laminar shear stress (63), which is mediated by p300 through acetylation of NF-kB and also increased H3Ac and H4Ac at the eNOS promoter following shear in HUVECs leading to increased eNOS expression (59). Although this study did not evaluate whether p300 and NF-kB directly interact at shear response elements in the eNOS promoter, it is likely that these factors may also interact at these elements to regulate eNOS expression. Together, these studies suggest that p300 cooperates with NF-kB to regulate eNOS expression implicating a role for chromatin modifiers in regulating the shear response through coordination of transcription factors. However, the *in vivo* role of such an interaction in ECs exposed to fluid shear stress in atherogenesis remains to be investigated.

In addition to disturbed flow, reactive oxygen species (ROS) also contribute to the pathogenesis of cardiovascular diseases (64). A significant source of ROS in the vasculature is the family of NADPH oxidases or Nox enzymes (65). These are oxidoreductases that have slightly different mechanisms of activation and roles. One Nox family member, Nox4, is the predominant isoform expressed in ECs. In HUVECs, expression of Nox4 is repressed through inhibition of HDACs with scriptaid (66). This leads to increased H4Ac and H3K27Ac, which prevents c-jun and RNA polymerase (RNAP II) from binding to the endogenous Nox4 promoter, thus decreasing Nox4 expression (66). These findings provide an example of how histone modifications are able to prevent binding of transcriptional machinery to promoter elements. Moreover, they provide further support to a less typical activating role for HDACs in regulating gene expression.

Indeed, the class II HDAC SirT1 also positively regulates antioxidant genes such as catalase and manganese superoxide dismutase in ECs *in vitro* (67). SirT1 deacetylates H4K16 when recruited to promoters of these antioxidant genes leading to increased promoter clearance by RNAP II despite its decreased binding to the proximal promoter. As with other HDACs, in addition to modifying histones when recruited to gene promoters, SirT1 also directly modified the transcription factors involved in regulating antioxidant genes, FoxO3a and PGC1α, to promote stabilization of a PGC1α–FoxO3a complex (67). Whether the ternary complex of SirT1-FoxO3a-PGC1α may then be recruited to antioxidant genes to upregulate their expression in response to oxidative stress remains to be investigated. SirT1-dependent association with and deacetylation of the FoxO family of transcription factors is also involved in regulation of angiogenesis (68). Together these studies demonstrate that modification of transcription factors while also being recruited by them to regulatory regions to modify chromatin structure is a common occurrence in regulation of gene expression. This is likely shared by many HATs and HDACs other than p300/CBP, HDACs, and SiRTs mentioned in this review and should be considered when investigating their role in cardiovascular disease. Moreover, the studies mentioned here discuss only some of the more commonly studied HATs and HDACs, but it is also likely that interaction of factors like GNAT or CLOCK with transcription factors is an essential component in gene regulation underlying cardiovascular pathogenesis.

## Histone Methylation in Cardiovascular Disease

### Histone Methylation and Myocardin, MRTFs, and SRF Interactions

In addition to cooperating with HATs and HDACs, the master regulators of muscle phenotype SRF, myocardin, and MRTFs also interact with histone methyltransferases and demethylases to regulate gene expression. SRF and other core cardiac transcription factors such as Tbx5 and Nkx2.5 interact with UTX, a H3K27-specific histone demethylase, to promote an open chromatin conformation and enhancer activation of target cardiac genes like ANF (Figure 1A) (18). Prevention of recruitment of UTX to enhancers of cardiac-specific genes using UTX null mice impaired cardiac differentiation, demonstrating the importance of this demethylase in regulating cardiac development. However, whether the loss of the specific interaction of UTX and Tbx5 or Nkx2.5 is primarily driving the impaired cardiac differentiation *in vivo* is unknown. Interaction of histone methylation and transcription factors in regulating development is not unique to cardiomyocytes. Indeed, the SMC-specific program of gene expression is activated by recruitment of WDR5, a necessary component of the SET/MLL family of methyltransferases, by SMC-selective pituitary homeobox 2 (69). WDR5 promotes expression of the SMC-specific gene program through mediating H3K4 methylation of SMC marker promoters. Together, these studies demonstrate a role for core and/or cell type-specific transcription factors in recruiting ubiquitously expressed histone methyltransferases and demethylases in control of lineage-specific gene expression.

SRF, myocardin, and MRTFs also mediate changes in gene expression in response to environmental stimuli through interactions with histone methyltransferases and demethylases. The histone demethylase JMJD2a cooperates with SRF/myocardin to upregulate expression of four-and-a-half LIM domains 1 (FHL1) (70), an important biomechanical stress sensor involved in cardiac hypertrophy (71). JMJD2a binds to the *Fhl1* promoter and downregulates H3K9 methylation in response to transverse aortic constriction (TAC) (70). JMJD2a and its activity likely enhance binding of SRF/myocardin to the *Fhl1* promoter, and SRF was required for JMJD2a-dependent activation of an *Fhl1* promoter reporter. JMJD2a knockdown attenuated hypertrophy following TAC (70). The authors hypothesize that JMJD2a interaction with SRF/myocardin may be a feed-forward loop in which SRF/myocardin initially recruits JMJD2a to the *Fhl1* promoter leading to downregulation of H3K9me3 and subsequent stabilization of SRF/myocardin binding. This study demonstrates the complexity in understanding whether chromatin modifiers or transcription factors recruit the other to gene regulatory sites and highlights the need for detailed mechanistic studies to understand these interactions in the regulation of gene expression.

**Figure 3:**
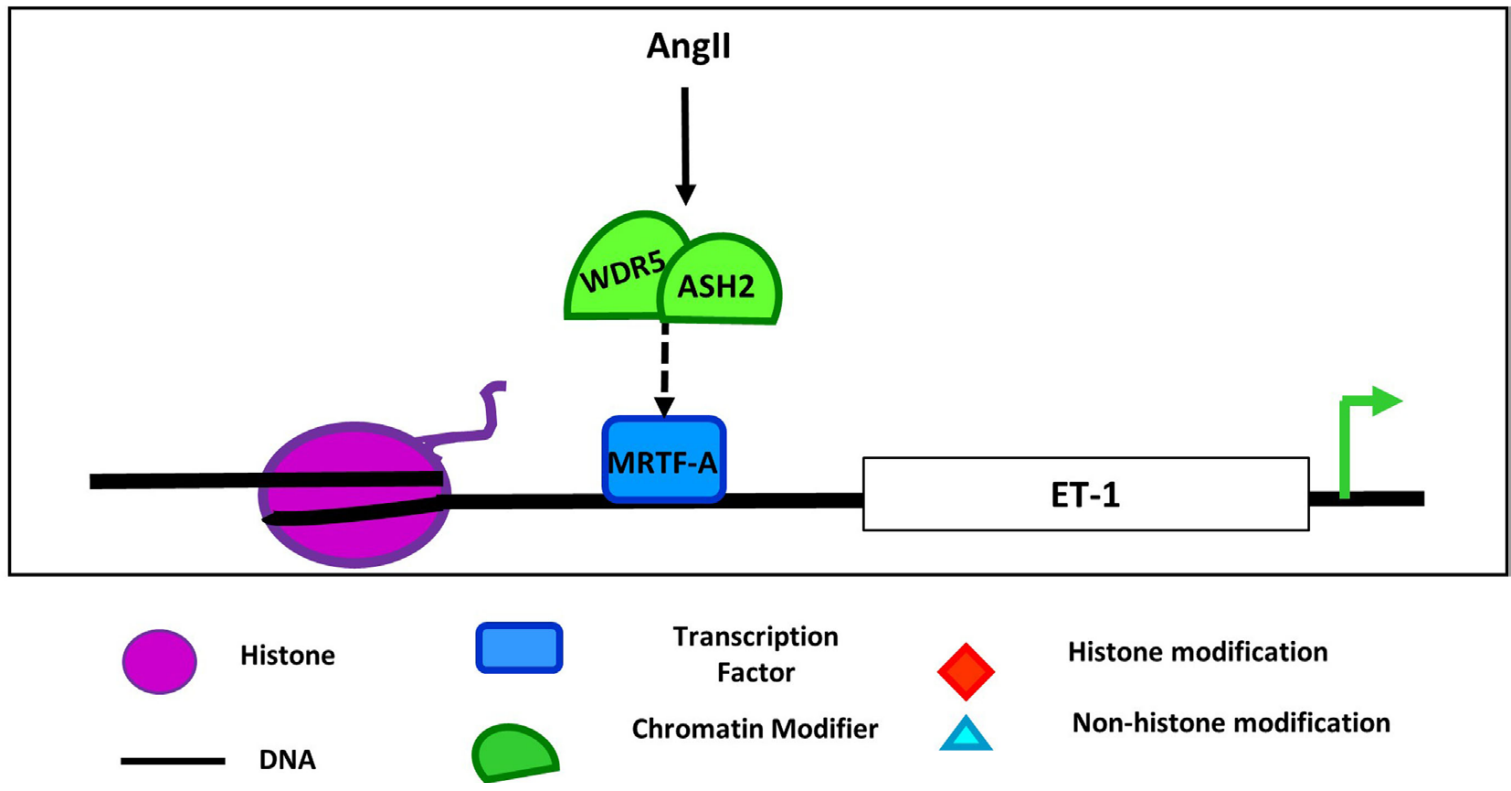
**Regulation of endothelin 1 by COMPASS components and MRTF-A in endothelial cell**. Angiotensin II (AngII) treatment induces the interaction of COMPASS components WDR5 and ASH2 with MRFT-A at the endothelin 1 promoter leading to upregulation of ET-1 expression (72).

In addition to interaction with demethylases, MRTFs also cooperate with methyltransferases to regulate gene expression. For example, in ECs, MRTF-A mediates the recruitment and interaction of the COMPASS components ASH2 and WDR5 and chromatin remodelers to the ET-1 promoter following AngII stimulation (Figure 3) (72). Recruitment of this complex promotes transcription of ET-1, which plays an important role in vasoconstriction, endothelial dysfunction, and development in numerous cardiovascular diseases (73). MRTF-A interaction with COMPASS and its components such as ASH2 is also important in regulation of inflammatory stimuli downstream of ET-1 (74), and LPS promoted the same MRTF-A-dependent recruitment of these COMPASS components to NF-kB target promoters in macrophages as demonstrated by ChIP and reChIP assays (75). Furthermore, knockdown of MRTF-A reduced the binding of COMPASS proteins demonstrating that, in this study, the transcription factor recruited the chromatin modifying proteins, but whether that is the case in all settings remains to be determined. Together, these findings demonstrate that the SRF, myocardin, and MRTF family of transcription factors mediate the recruitment or are potentially recruited by both demethylases and methyltransferases in numerous cellular contexts suggesting widespread applicability of the role of these interactions in cardiovascular pathologies. Furthermore, as SRF/myocardin are ubiquitously expressed proteins involved in the regulation of cytoskeletal genes, there are likely cell type-specific and locus-specific interactions between them and epigenetic regulators.

### Histone Methylation and Vascular Homeostasis

Endothelial homeostasis is also regulated by cooperation of transcription factors and histone methyltransferases and demethylases. KLF2 is an important EC transcription factor that promotes an anti-inflammatory and antithrombotic surface through regulation of numerous genes including eNOS and thrombomodulin (76). Laminar flow activates MEF2, which promotes transcription of KLF2. The proatherogenic stimulus low-density lipoprotein (LDL) alters binding interactions at the *KLF2* promoter, decreasing MEF2 binding while increasing occupation by the epigenetic reader, MECP2, and H3K27 histone methyltransferase, EZH2, leading to repressed KLF2 expression (77). As the binding sites for these factors were overlapping or all within a 169 base pair promoter region, these findings suggest that these epigenetic factors can competitively bind to promoters to inhibit transcription factor binding and subsequent gene activation.

Endothelial cells are sensitive to hypoxia, a key stimulus for angiogenesis. Transcription factor-facilitated chromatin interactions with epigenetic modifiers are also observed with hypoxia-dependent upregulation of the glucose transporter, GLUT3, in HUVECs. Expression of this gene was increased through HIF1-α-dependent recruitment of the demethylase KDM3A and subsequent demethylation of H3K9 at the transcriptional start site and enhancer regions of GLUT3 (*SLC2A3*) (78). HIF1-α and KDM3a coimmunoprecipitated, and knockdown of HIF1-α prevented binding of KDM3A to regulatory sites upstream of GLUT3. Therefore, HIF1-α and KDM3a cooperate to increase GLUT3 expression and subsequent glucose uptake, which is important to maintain energy availability under hypoxic conditions.

In addition to histone lysine methylation, histone arginine methylation also influences transcription factor recruitment during SMC phenotypic switching in atherogenic conditions (79). Smooth muscle-specific knockout of the Wnt co-receptor, LDL receptor-related protein (LRP6), in LDL knockout mice fed a high-fat diet, resulted in increases in osteochondrogenic genes like osteopontin (OPN) and vascular calcification. Increased OPN expression was mediated by protein arginine methyltransferase 4 (PRMT4)-dependent dimethylation of Arg-17 on histone H3. This H3R17me2 mark recruits the transcription factor USF1, leading to activation of the OPN promoter. Recruitment of USF1 and induction of OPN expression were inhibited by the arginine demethylase JMJD6 (79). Although this study elucidated a role for PRMT4 and USF1 cooperation in upregulating OPN expression in the absence of LRP-mediated Wnt signaling, it is possible that these factors also control OPN upregulation in other instances of vascular calcification. Together, these findings demonstrate that histone methylating and demethylating enzymes cooperate with a variety of transcription factors in numerous cellular contexts to regulate gene expression.

## DNA Methylation in Cardiovascular Disease

### DNA Modifications and Transcription Factor Interactions

Aberrant expression of DNMTs, TETs, or their subsequent DNA modifications is involved in various cardiovascular diseases (11, 80). However, the potential role of interactions between these enzymes or their marks and transcription factors in the molecular pathogenesis of these diseases are less well understood. DNA methylation appears to have a pro-inflammatory role (81) and is important in regulation of endothelial cell-specific expression of eNOS (82). DNA methylation of the *eNOS* promoter in non-EC types prevents binding of the transcription factors Sp1, Sp3, and Ets1, whereas hypomethylation of this promoter in ECs allows for these factors to drive its expression (83). Interestingly, eNOS expression is also regulated through aberrant DNA methylation of the promoters of transcription factors like KLF2 and KLF4 that bind and activate the eNOS promoter (77, 84). Disturbed flow, associated with atheroprone regions of the vasculature (85), increased the methylation of the *KLF4* promoter by DNMT3a. This methylation precludes binding of the transcription factor MEF2, a key driver of KLF4 expression. The resulting decrease in KLF4 expression subsequently reduces eNOS expression in ECs (84).

### DNA Methylation Modifiers and Transcription Factor Interactions

While these eNOS regulation studies suggest a role for DNA methylation in preventing transcription factor binding to promoters, to our knowledge, direct interaction of DNA methylating and demethylating enzymes with transcription factors to regulate gene expression in the context of cardiovascular diseases has not been investigated. However, there is evidence that transcription factors can guide DNMTs and TETs to particular promoter regions in other cell types including bone marrow-derived dendritic cells, neurons, and adipocytes (86–89). For example, during inflammation resolution, TET2 is recruited to *Il-6* promoter by IκBζ in bone marrow-derived dendritic cells to repress IL-6 expression independent of TET2 enzymatic activity (86). IL-6 is also proatherogenic by promoting inflammatory cell infiltration into the plaque, endothelial dysfunction, and VSMC proliferation, and uptake of LDL (90). TET2 has recently been implicated as a master epigenetic regulator of SMC phenotype, and its expression is inversely correlated with severity of atherosclerosis in human plaques (91). A recent study revealed an opposing relationship between TET2 and DNMT1 in SMCs, with DNMT1 leading to methylation of the TET2 promoter, suppressing expression of TET2 and myocardin. Furthermore, DNMT inhibitors suppressed both atherosclerosis and intimal hyperplasia (92). While these factors regulate expression of KLF4 and myocardin (91, 92), whether and how TET2 and DNMTs interact with SMC-specific transcription factors is not yet known but will be important to determine. These studies demonstrate that DNA-modifying enzymes associate with transcription factors in numerous cell contexts, suggesting that perhaps similar associations occur to regulate gene expression in the pathogenesis of cardiovascular disease.

## Other Histone Modifiers and Cardiovascular Disease

DNA methylation, histone methylation, and histone acetylation along with the enzymes that make these marks are the most commonly studied epigenetic regulators. However, there are also several other types of modifications and enzymes that have yet to be studied. Histone phosphorylation is commonly used as a marker of cell division although it also plays a role in DNA repair, chromatin compaction during cell division, apoptosis, and even regulation of transcription (93). As with phosphorylation of other proteins, there are numerous enzymes that phosphorylate and dephosphorylate histones, but few have been studied for their potential role in cardiovascular disease pathogenesis. Similar to histone phosphorylation, histone ubiquitination is regulated by numerous ubiquitin ligases and deubiquitinating enzymes and is also involved in many processes including DNA repair and transcriptional regulation (94, 95). Although it is known that dysregulated ubiquitination of proteins is involved in cardiovascular disease development and progression (96, 97), the potential roles of histone ubiquitination or the enzymes that add or remove this mark are unclear. It has been shown that ubiquitin-proteasome-mediated degradation of histone variants is upregulated during cardiac hypertrophy (98). Moreover, in congenital heart disease, there are *de novo* mutations in genes involved in ubiquitination of histones (99), suggesting that histone ubiquitination might be important in cardiovascular disease pathology. Another histone modification less commonly studied is O-linked β-*N*-acetylglucosamine (O-GlcNAc). A role for O-GlcNAcylated non-histone proteins, along with the enzymes that catalyze the addition and removal of it, is well established in cardiovascular pathologies (100). However, few substrates that mediate these pathologies have been identified, and the role of O-GlcNAc-modified histones in cardiovascular disease is unknown. Studies on these enzymes and others, including chromatin remodeling enzymes, are necessary to better understand cardiovascular disease pathogenesis at a mechanistic level.

## Summary and Perspectives

Cardiovascular diseases continue to be a major health concern in the United States and worldwide. Although considerable progress has been made in understanding the cellular and molecular mechanisms underlying cardiovascular disease pathology, few studies have investigated how transcription factors and chromatin modifiers including DNA methylation modifiers, histone methylation enzymes, and histone acetylation modifiers cooperate to regulate gene expression underlying cardiovascular disease. While this area of investigation is still relatively underdeveloped due to challenges in methodology described below, it is becoming clear that control of gene expression is a coordinated effort between transcription factors and chromatin remodeling enzymes. There are several classical modes in which these factors can influence each other’s activity. These include the following: (1) DNA or histone modifications prevent or induce transcription factor binding (e.g., H4Ac and H3K27Ac preventing c-jun and RNAP II binding in Section “Histone Acetylation and ECs”). (2) HATs or HDACs posttranslationally modify transcription factors to alter their binding or specificity (e.g., p300 and HDAC2 modification of GATA4 in Section “Histone Acetylation and Cardiovascular Development”). (3) Chromatin modifiers are recruited to (or excluded from) specific loci by transcription factors (e.g., MRTF-A recruitment of COMPASS in Section “Histone Methylation and Myocardin, MRTFs, and SRF Interactions”). (4) Transcription factors are recruited to (or excluded from) specific loci by the presence of a chromatin modifying enzyme (e.g., JMJD2a and SRF/myocardin in Section “Histone Methylation and Myocardin, MRTFs, and SRF Interactions”). In this review, we have summarized examples of these paradigms that have been implicated in specific gene regulation in systems relevant to cardiovascular disease. Many of the studies, however, have reported an interaction between a transcription factor and epigenetic modifier without demonstrating that the specific interaction (or lack thereof) is *necessary* for cardiovascular disease progression. This level of evidence can be difficult to achieve with current methods (see below). However, the importance of these interactions in cardiovascular disease is being realized and will likely be further developed in the future as methods improve.

Although further studies are necessary to better understand the intersection between transcriptional and epigenetic regulation of gene expression in cardiovascular disease, conducting these studies proves to be challenging for three main reasons. First, several of these chromatin modifying proteins, particularly HATs and HDACs, have roles in modifying non-histone proteins like transcription factors. Therefore, it remains difficult to elucidate whether effects on gene expression are due to direct interaction of these proteins to modify the transcription factor itself or interaction for recruitment and modulation of chromatin structure. Second, chromatin-modifying proteins often work in larger complexes or recruit other epigenetic factors that complicate determining how factors interact to regulate gene expression. Finally, many of the techniques currently used in these studies do not always sufficiently address whether one factor recruits another or how they interact to modify control gene activation or repression. Techniques to detect colocalization of transcription factors and epigenetic modifiers at gene loci [e.g., ChIP-reChIP (101, 102)], to overcome cellular heterogeneity by investigating chromatin changes at the single-cell level [e.g., single-cell ChIP-Seq (103)], or to detect what is bound at a specific locus in an unbiased manner [e.g., reverse ChIP (104)] have been developed. However, these methods have technical limitations including amount of sample required, low yields, and high costs. There is a need to continue to improve upon these methods to allow their more widespread use in the field. Therefore, many studies, including many described in this review, perform a single ChIP to demonstrate binding of factors individually to similar regions followed by separate coimmunoprecipitation experiments documenting the interaction of these factors (which may or may not occur at the same location in chromatin). Ideally, studies would demonstrate that specific disruption of interaction between these factors prevents downstream gene transcription and the functional readout or they would perform sequential chromatin immunoprecipitation experiments (ChIP-reChIP) to show that these transcription factors and chromatin modifiers are colocalized upstream of a particular gene. Although, incorporating knockdown of either factor could demonstrate that binding of one determines binding of the other, use of knockdown or overexpression techniques leads to difficulty in determining if observed outcomes are due to direct or indirect effects of the factors interacting. While assessing interactions between endogenous factors *in vivo* would be ideal, transcription factors and chromatin modifiers are oftentimes expressed at low levels or there is a lack of reliable reagents. As such, epigenetic studies commonly employ *in vitro* methods including use of exogenous promoters or overexpression of factors. However, as culturing cells *in vitro* alone has been shown to alter the epigenome (105), studies primarily employed *in vitro* have several limitations and may not be an accurate representation of what occurs in the setting of cardiovascular disease. Therefore, a combination of studies using *in vivo* methods, primary cells, and endogenous promoters and expression of factors will be important in elucidating the relevance of these interactions in cardiovascular disease.

With the realization of the complexity, but necessity, of studying the interaction of transcription factors and epigenetic modifiers in regulation of gene expression, and continued improvements in necessary techniques, several important questions should be addressed. As previously described, the roles of other chromatin-modifying proteins and identification of new transcription factor–epigenetic regulator interactions in pathogenesis of cardiovascular disease remain an important area of investigation. In addition, many studies focus on the interaction of the proteins at promoter regions to regulate gene expression, but such interactions at enhancer elements, repressors, or elsewhere remain largely unexplored. Perhaps most importantly, the mechanisms that govern the locus specificity of these interactions is of significant interest given that many of the epigenetic modifiers and transcription factors are ubiquitously expressed. Perhaps, in some cases, binding motif sequence specificity of transcription factors can account for the locus-specific targeting of some epigenetic regulators if these factors interact to regulate gene expression. Conversely, DNA or histone modifications within a specific sequence may recruit transcription factors to a locus to regulate gene expression. However, these instances do not account for all situations and do not always address why these specific interactions occur at a single locus following a given stimulus. It is likely that signaling cascades generated by extracellular stimuli converge on these transcriptional and epigenetic regulators in a context-specific manner, combined with a cohort of tissue-restricted proteins to ultimately determine tissue- and disease-specific gene regulation. Finally, this area of investigation is important as genome-wide association studies (GWAS) have identified many SNPs associated with cardiovascular disease. As most variants identified by GWAS lie outside of protein-coding regions (106), it is likely that many of these SNPs may reside in as yet-unidentified regulatory elements where transcription factors and chromatin modifiers interact. Addressing these questions will provide a better understanding of the mechanisms underlying cardiovascular disease pathogenesis that will allow the development of better diagnostic tools and therapeutics to address this global health burden.

## Author Contributions

AB researched, wrote, and edited the manuscript. KM wrote and edited the manuscript.

## Conflict of Interest Statement

The authors declare that the research was conducted in the absence of any commercial or financial relationships that could be construed as a potential conflict of interest.
